# An Optimization-Based Family of Predictive, Fusion-Based Models for Full-Reference Image Quality Assessment

**DOI:** 10.3390/jimaging9060116

**Published:** 2023-06-08

**Authors:** Domonkos Varga

**Affiliations:** Ronin Institute, Montclair, NJ 07043, USA; domonkos.varga@ronininstitute.org

**Keywords:** full-reference image quality assessment, optimization, quality-aware features

## Abstract

Given the reference (distortion-free) image, full-reference image quality assessment (FR-IQA) algorithms seek to assess the perceptual quality of the test image. Over the years, many effective, hand-crafted FR-IQA metrics have been proposed in the literature. In this work, we present a novel framework for FR-IQA that combines multiple metrics and tries to leverage the strength of each by formulating FR-IQA as an optimization problem. Following the idea of other fusion-based metrics, the perceptual quality of a test image is defined as the weighted product of several already existing, hand-crafted FR-IQA metrics. Unlike other methods, the weights are determined in an optimization-based framework and the objective function is defined to maximize the correlation and minimize the root mean square error between the predicted and ground-truth quality scores. The obtained metrics are evaluated on four popular benchmark IQA databases and compared to the state of the art. This comparison has revealed that the compiled fusion-based metrics are able to outperform other competing algorithms, including deep learning-based ones.

## 1. Introduction

With social media and streaming applications booming, it is required from systems, that are able to quickly transmit a large number of images, to provide the best available user experience [1]. However, various distortions are added to digital images during storage, compression, and transmission. Therefore, the continuous evaluation and monitoring of image quality is of great importance to content providers [2]. As a consequence, objective image quality assessment (IQA) has become a very hot research topic [3], because it tries to devise mathematical models that are able to give perceptual quality estimation consistent with human judgment. The literature usually divides objective IQA into three branches [4,5] based on the availability or unavailability of the reference (distortion-free) images in the quality evaluation process. As the terminology suggests, full-reference (FR) IQA evaluates the quality of distorted images with full access to their reference counterparts, while no-reference (NR) IQA has no access and reduced-reference (RR) IQA has partial access to them.

Because the underlying model of the human visual system (HVS) is extremely complex and its many elements are not fully understood [6], researchers have proposed many FR-IQA algorithms, which take into consideration different aspects of the HVS, over the years. Recently, there have been numerous attempts to increase the performance of FR-IQA by combining several already existing FR-IQA metrics to compile a “super” evaluator. First, Okarma [7] introduced such a fusion-based metric by applying the product and power of MS-SSIM [8], VIF [9], and R-SVD [10]. Later, this idea was developed further into several directions. A line of works utilized optimization or regression techniques to determine optimal weights or exponents in summations or products of already existing FR-IQA metrics. For instance, Oszust [11] determined the optimal weights using a genetic algorithm with a root mean square error (RMSE) objective function which was calculated between predicted and ground-truth scores. Bakurov et al. [12] chose a similar solution, but the authors revisited the SSIM [13] and MS-SSIM [8] metrics to find optimal parameters in their formulas, using evolutionary and swarm intelligence methods instead of the originally proposed grid search. On the other hand, Okarma [14] used the MATLAB fminsearch function to determine the optimal exponents in a weighted product of traditional FR-IQA metrics. Another line of works utilize machine learning techniques to create fusion-based methods. The illustrative example is the paper of Lukin et al. [15] where the results of traditional FR-IQA metrics were used as a feature vector to train a shallow neural network. Amirshahi et al. [16] compiled a fusion-based metric by comparing the activation maps produced through reference and distorted images of an AlexNet [17] convolutional neural network using traditional image quality metrics.

### 1.1. Contributions

In this paper, we make the following contributions. We propose a novel framework for FR-IQA that combines multiple metrics and tries to leverage the strength of each by formulating FR-IQA as an optimization problem. Following the idea of other fusion-based metrics, the perceptual quality of a test image is defined as the weighted product of several already existing, hand-crafted FR-IQA metrics. Unlike other methods [7,18,19], the weights in the product are determined in a novel optimization-based framework and the objective function is defined to maximize the correlation strength and minimize the root mean square error between the predicted and ground-truth quality scores.

### 1.2. Structure of the Paper

To provide a clear and organized presentation of our work, this paper is structured as follows. In Section 2, we discuss the main approaches of FR-IQA and summarize significant methods of the field. Section 3 outlines our proposed method. In Section 4, we present the experimental results and analyze the performance of our method by comparing it to other state-of-the-art methods. We conclude this paper in Section 5 and discuss potential applications and future research directions.

## 2. Related Work

Taking the mean square error between reference and distorted images is a simple and straightforward FR-IQA metric. However, the provided quality scores do not correlate well with human judgment [20]. Similarly, PSNR [21] is also simple and straightforward but fails to give satisfactory results [22]. Other metrics take the sensitivity of the HVS to structural degradation into consideration, such as the structural similarity index (SSIM) [13]. On the basis of SSIM [13], a large number of FR-IQA metrics has been proposed over the years, such as MS-SSIM [8], CW-SSIM [23], ESSIM [24], GSSIM [25], IW-SSIM [26], and 3-SSIM [27]. In SSIM [13], a comparison between the distorted and reference (distortion-free) images is performed on the basis of three features, i.e., luminance, contrast, and structure. To be more specific, the SSIM between two images (denoted here by **A** and **B**) in an image patch around (x,y) coordinates is given as

(1) $$SSIM\left(x,y\right)={\left(l(x,y)\right)}^{\alpha }\times {\left(c(x,y)\right)}^{\beta }\times {\left(s(x,y)\right)}^{\gamma },$$

where the luminance component is defined as

(2) $$l\left(x,y\right)=\frac{2\mu_{A}\left(x,y\right)\mu_{B}\left(x,y\right)+C_{1}}{{\left(\mu_{A}\left(x,y\right)\right)}^{2}+{\left(\mu_{B}\left(x,y\right)\right)}^{2}+C_{1}},$$

the contrast component is given as

(3) $$c\left(x,y\right)=\frac{2\sigma_{A}\left(x,y\right)\sigma_{B}\left(x,y\right)+C_{2}}{{\left(\sigma_{A}\left(x,y\right)\right)}^{2}+{\left(\sigma_{B}\left(x,y\right)\right)}^{2}+C_{2}},$$

and the structure component is determined as

(4) $$s\left(x,y\right)=\frac{\sigma_{AB}\left(x,y\right)+C_{3}}{\sigma_{A}\left(x,y\right)\sigma_{B}\left(x,y\right)+C_{3}}.$$

In Equations (1)–(4), $\mu_{A}\left(x,y\right)$ and $\mu_{B}\left(x,y\right)$ denote the average of the pixel values in the image patch around (x,y) in images **A** and **B**, respectively. Similarly, ${\left(\sigma_{A}\left(x,y\right)\right)}^{2}$ and ${\left(\sigma_{B}\left(x,y\right)\right)}^{2}$ stand for the variances. Further, $\sigma_{AB}\left(x,y\right)$ is the covariance calculated between the two images from **A** and **B**. The constants are calculated as $C_{1}={\left(K_{1}L\right)}^{2}$, $C_{2}={\left(K_{2}L\right)}^{2}$, and $C_{3}=C_{2}/2$. Further, *L* stands for the dynamic range of the pixel values, and for images with 8-bit depth, L=255 is the recommended value. By default, $K_{1}=0.01$ and $K_{2}=0.03$ are also constants whose exact values were chosen by Wang et al. [13] after an ablation study. To give the perceptual quality of the distorted image in possession of the reference image, the arithmetic average of SSIM(x,y) is taken. As already mentioned, a huge number of FR-IQA metrics has been inspired by the original SSIM. For comprehensive overviews on SSIM-motivated methods, the following papers can be recommended [12,28,29,30,31]. Here, several representative methods are mentioned in the following. The authors of multi-scale SSIM [8] extended the idea of SSIM into multiple scales. Sampat et al. [23] replaced the components of SSIM by complex wavelet coefficients [32]. In contrast, Zhang et al. [24] defined an edge strength-based image quality metric where the strength of edges was defined in horizontal and diagonal directions using directional derivatives. Chen et al. [25] took a similar approach, but the edge information was characterized by gradient magnitudes. In [26], the authors used the information content measure as a weighting factor in the pooling process of SSIM [13] to obtain improved prediction results. This idea was further improved by Larson et al. [33] where low-level distortions, which are nearly imperceptible, were modeled by local luminance and contrast masking, while high-level distortions were modeled using spatial-frequency components. Kolaman and Yadid-Pecht [34] extended the SSIM metric to colorful images by modeling colors with quaternions. In [35], the authors analyzed different strategies aiming at the usage of visual saliency maps [36] in improving IQA algorithms. A proposal was the weighting of local estimates by local saliency values. In [37], first- and second-order Riesz-transform [38] coefficients were used to create feature maps for the reference and the distorted images which were compared to give an estimation of the perceptual image quality. Similarly, Zhang et al. [39] compared feature maps to quantify image quality, but the authors used phase congruency [40] and gradient magnitude maps.

Recently, the scientific community has paid more and more attention to the deployment of machine and deep learning models in almost all computer vision tasks [41]. The field of image quality assessment has accommodated this trend [3,42]. For instance, Tang et al. [43] extracted spatial and frequency domain features from reference–distorted image pairs and trained a random forest regressor for image quality prediction. In contrast, Bosse et al. [44] devised a convolutional neural network (CNN) architecture which can be trained end-to-end on single images or on image pairs. Similarly, Zhang et al. [45] trained an end-to-end CNN in a patch-wise fashion for FR-IQA and compared the effectiveness of deep features extracted from different pretrained CNNs. As a consequence, it can be used for both NR- and FR-IQA. In [46], the authors proposed a pairwise-learning framework for FR-IQA. Several works extracted deep features via pretrained CNNs from reference–distorted image pairs and compared them to assess the perceptual image quality. For instance, Amirshahi et al. [47] compared the histograms of deep features using a histogram intersection kernel (HIK) [48] at multiple levels. The perceptual quality was obtained by aggregating the similarity scores provided by the HIKs. Later, this approach was further developed in [16] by replacing the HIK in comparing convolutional feature maps with a traditional image similarity metric. In [49], the authors used the error map calculated between the reference and distorted images and the subjective saliencies of the distorted images to train a CNN for perceptual image quality estimation.

Recently, several researchers devised fusion-based FR-IQA methods where the goal is creating a “super-evaluator" using already known FR-IQA metrics to achieve advanced performance. A large number of fusion-based algorithms determine weights for each FR-IQA metric in a summation or in a product of sequence [1]. An illustrative example is the method proposed by Okarma [7]. Namely, the properties of three different FR-IQA metrics were examined thoroughly, and a combined metric was devised based on the metrics’ arithmetical product and power. By using mathematical optimization techniques, the parameter values of this fusion-based metric were refined in [14]. Oszust [50] and Yuan et al. [51] also developed this approach further by applying lasso regression and kernel ridge regression, respectively. Oszust [11] determined the weights in a linear combination of traditional FR-IQA metrics by applying a genetic algorithm. In [52], this approach was further developed by using multi-gene genetic programming [53]. The effectiveness of this approach was also demonstrated on screen content images [54]. Simulated annealing was also applied in this framework [55]. Machine learning techniques were also used in creating fusion-based algorithms. An illustrative example is Lukin et al.’s [15] work. Namely, the authors used the outcomes of several FR-IQA metrics as features and trained a neural network on top of them to predict perceptual quality. A similar approach using a neural network was proposed for the quality assessment of remote sensing images [56].

In summary, this section has highlighted the various approaches that have been proposed in the literature for FR-IQA. Although the reviewed studies have contributed significantly to the field, a detailed overview about IQA or FR-IQA is out of the scope of this study. For a general overview about the field of IQA, the PhD dissertations of Jenadeleh [57] and Men [58] can be recommended while Min-juan et al. [59], Phadikar et al. [60], George et al. [61], and Pedersen et al. [30] provide state-of-the-art studies on FR-IQA.

## 3. Proposed Method

In [7], Okarma took into account the different properties of three different FR-IQA metrics (MS-SSIM [8], VIF [62], and R-SVD [10]) and defined a combined quality metric (CQM):

(5) $$CQM={\left(MSSSIM\right)}^{a}\times {\left(VIF\right)}^{b}\times {\left(RSVD\right)}^{c},$$

where a=7, b=0.3, and c=−0.15 values were used because they lead to a near-optimal solution on an IQA benchmark database. Following this basic idea of Okarma [7], a fusion-based metric is defined as the weighted product of *n* different traditional FR-IQA methods’ results:

(6) $$Q_{p}=\prod_{i=1}^{n}q_{i}^{\alpha_{i}},$$

where $q_{i}$s are the results of the applied FR-IQA metrics and $\alpha_{i}$s are the associated weights. Specifically, we chose n=18 and the following metrics were utilized: FSIM [39], FSIMc [39], GSM [63], IFC [9], IW-SSIM [26], MAD [33], MS-SSIM [8], NQM [64], PSNR [21], RFSIM [37], SFF [65], SSIM [13], SR-SIM [66], UQI [67], VIF [62], VSI [68], and VSNR [69]. A summary of the acronyms of the used FR-IQA metrics can be found in Table 1. In the literature, the parameters of an FR-IQA metric are tuned on a smaller subset of images. In the case of a traditional metric, such as SSIM [13] given by Equations (1)–(4), the number of tunable parameters is one or two. As a consequence, appropriate values can be easily found applying for cycles over a search space. In contrast, our fusion-based metric given by Equation (6) contains n=18 parameters and an optimization task is defined to find their exact values. To determine the optimal weights (parameters) in Equation (6), the following optimization problem is defined:

(7) $$\begin{array}{c} \max_{\alpha }\frac{SROCC\left(Q_{p},S\right)+KROCC\left(Q_{p},S\right)}{RMSE(F\left(Q_{p},\beta \right),S)},\\ \mathrm{subject}\;\mathrm{to}\;\alpha_{i}\in R,n\in N,\beta \geq 0, \end{array}$$

where $Q_{p}$ and S are vectors containing the predicted and ground-truth quality scores, respectively. SROCC(·,·) and KROCC(·,·) denote the Spearman’s rank-order correlation coefficient and Kendall’s rank-order correlation coefficient calculated between two vectors, respectively. Further, RMSE(·,·) is the root mean square error determined between two vectors. Prior to the calculation of the RMSE, a non-linear mapping is applied to the predicted scores following the recommendations of [4]. In this paper, the following non-linear function was applied

(8) $$F\left(Q_{p},\beta \right)=\beta_{1}\left(\frac{1}{2}-\frac{1}{1+e^{\beta_{2}\left(Q_{p}-\beta_{3}\right)}}\right)+\beta_{4}Q_{p}+\beta_{5},$$

with the following β parameters, $\beta_{1}=10$, $\beta_{2}=0$, $\beta_{3}=mean\left(Q_{p}\right)$, $\beta_{4}=1$, and $\beta_{4}=0.1$, which were also used in the MATLAB implementation of the VSI [68] method.

Because an FR-IQA metric is supposed to provide objective scores which have a high correlation and low RMSE with respect to subjective quality scores collected from human observers, the objective function’s—given by Equation (7)—numerator consists of the sum of SROCC and KROCC while the denominator corresponds to the RMSE. Our preliminary investigations revealed that considering only SROCC or KROCC may result in a higher RMSE than those of the state of the art. That is why we decided to divide the sum of SROCC and KROCC by the RMSE.

**Figure 1:**
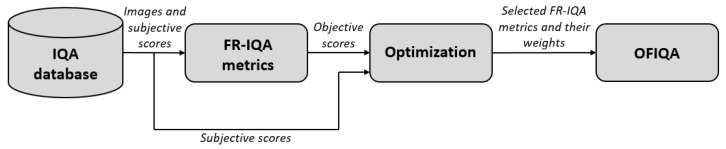
Twenty percent of the reference images with their corresponding distorted counterparts are selected to determine the parameters of the proposed fusion-based metric in the optimization process. The resulting metric is codenamed as OFIQA.

**Figure 2:**
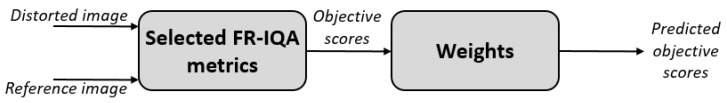
The weighted product of the selected FR-IQA metrics is used to estimate the perceptual quality of a distorted image in the evaluation stage.

Two nature-inspired optimization methods, such as the genetic algorithm [70] (GA) and pattern search [71] (PS), were applied to the problem defined by Equation (7) to determine the optimal weights. Further, the simplex method of Lagarias et al. [72], which is implemented in the *fminsearch* function of MATLAB’s Optimization Toolbox, was also used. To improve the efficiency of the fusion, each method was able to execute model selection (which FR-IQA metric to aggregate or not). The main motivation behind the choice of optimization methods was to collect algorithms that are able to give at least approximate solutions for NP (non-deterministic polynomial-time) hard problems. Figure 1 and Figure 2 depict the compilation of the proposed fusion-based FR-IQA metric. Specifically, the fusion was carried out on 20% of the reference images and their corresponding distorted counterparts in our method. In the literature, 20% is a common choice for parameter setting in a derived formula [73,74], but there are also researchers who used 30% [39] or 80% [75]. In total, four fusion strategies were realized with the help of one optimization method and benchmark database. Further, the fusion strategies were also cross-database tested. Each optimization method was carried out 100 times and the best solution was finally selected. We codenamed our method OFIQA to refer to the fact that the decision fusion was carried out via optimization.

In the GA, the population size and the number of generations were set to 100. The best solutions on the four benchmark databases were provided by the following equations:

(9) $$OFIQA_{LIVE}^{GA}=FSIM^{-3.6888}\times GSM^{12.5693}\times IWSSIM^{0.9556}\times IFS^{-1.8159},$$

(10) $$\begin{array}{c} OFIQA_{TID2013}^{GA}=VSI^{13.9336}\times FSIMc^{2.2946}\times GSM^{-10.864}\times NQM^{-0.1713}\times \\ \times SRSIM^{2.4651}\times IFS^{0.5139}, \end{array}$$

(11) $$\begin{array}{c} OFIQA_{TID2008}^{GA}=VSI^{7.0221}\times FSIM^{0.259}\times FSIMc^{1.0055}\times GSM^{-19.8267}\times \\ \times PSNR^{0.1471}\times VIF^{0.1452}\times SFF^{2.4029}, \end{array}$$

(12) $$\begin{array}{c} OFIQA_{CSIQ}^{GA}=FSIMc^{-2.7532}\times MAD^{0.9692}\times MSSSIM^{1.1892}\times SSIM^{1.6561}\times VIF^{-0.75}\times \\ \times IFS^{-3.4013}\times SFF^{2.2901}. \end{array}$$

In the case of the PS after 100 runs, the following fusion-based metrics can be obtained:

(13) $$\begin{array}{c} OFIQA_{LIVE}^{PS}=FSIM^{0.6964}\times FSIMc^{-2.6056}\times MAD^{1.0817}\times MSSSIM^{-0.4711}\times \\ \times SSIM^{0.7302}\times UQI^{0.9946}, \end{array}$$

(14) $$\begin{array}{c} OFIQA_{TID2013}^{PS}=VSI^{24.1037}\times FSIM^{0.9292}\times GSM^{-19.5555}\times IWSSIM^{2.1053}\times \\ \times MSSSIM^{-6.1562}\times PSNR^{0.4649}\times VIF^{0.5463}\times SFF^{4.3998}, \end{array}$$

(15) $$\begin{array}{c} OFIQA_{TID2008}^{PS}=VSI^{23.5097}\times FSIM^{1.2155}\times FSIMc^{0.2494}\times GSM^{-21.8595}\times \\ \times IWSSIM^{1.2984}\times MSSSIM^{-1.9792}\times PSNR^{0.5571}\times SSIM^{-1.8374}\times \\ \times VIF^{0.5491}, \end{array}$$

(16) $$\begin{array}{c} OFIQA_{CSIQ}^{PS}=FSIM^{0.3471}\times FSIMc^{0.7575}\times GSM^{-60.4948}\times MAD^{1.8509}\times \\ \times NQM^{0.8049}\times PSNR^{0.8181}\times UQI^{0.5142}\times VIF^{-0.1294}. \end{array}$$

Using the method of Lagarias et al. [72], the following fusion metrics can be obtained:

(17) $$\begin{array}{c} OFIQA_{LIVE}^{fmin}=VSI^{0.442}\times FSIMc^{1.1986}\times GSM^{1.0479}\times IFC^{-0.1531}\times \\ \times IWSSIM^{1.8895}\times MAD^{1.6539}\times MSSSIM^{-0.8459}\times NQM^{0.4463}\times \\ \times PSNR^{-0.2781}\times RFSIM^{-0.1719}\times SRSIM^{-1.1448}\times SSIM^{0.0811}\times \\ \times UQI^{0.0955}\times VIF^{-0.6669}\times VSNR^{0.0765}\times SFF^{-0.1433}. \end{array}$$

(18) $$\begin{array}{c} OFIQA_{TID2013}^{fmin}=VSI^{0.6577}\times FSIM^{0.1742}\times FSIMc^{0.7197}\times GSM^{0.6092}\times \\ \times IFC^{0.3768}\times IWSSIM^{0.8904}\times MAD^{0.4482}\times MSSSIM^{0.9852}\times \\ \times NQM^{0.6304}\times PSNR^{0.8077}\times RFSIM^{0.3861}\times SSIM^{0.7985}\times \\ \times UQI^{0.3612}\times VIF^{0.0149}\times VSNR^{0.3338}\times IFS^{0.8979}\times \\ \times SFF^{0.9113}, \end{array}$$

(19) $$\begin{array}{c} OFIQA_{TID2008}^{fmin}=VSI^{2.0607}\times FSIM^{0.4211}\times FSIMc^{0.8323}\times GSM^{0.1672}\times \\ \times IFC^{0.0322}\times MAD^{0.0753}\times NQM^{0.098}\times PSNR^{0.3727}\times \\ \times RFSIM^{0.5523}\times SRSIM^{0.5783}\times SSIM^{-0.2377}\times UQI^{-0.3083}\times \\ \times VIF^{0.5273}\times VSNR^{-0.0292}\times IFS^{1.9221}\times SFF^{0.0902}, \end{array}$$

(20) $$\begin{array}{c} OFIQA_{CSIQ}^{fmin}=VSI^{-0.4328}\times FSIM^{0.4287}\times GSM^{0.0521}\times IFC^{0.0569}\times \\ \times IWSSIM^{-1.4847}\times MAD^{0.982}\times MSSSIM^{0.3763}\times NQM^{0.3919}\times \\ \times PSNR^{0.2141}\times RFSIM^{-0.1544}\times SRSIM^{0.7473}\times UQI^{0.2373}\times \\ \times VIF^{-0.2373}\times VIF^{-0.2243}\times VSNR^{0.1147}\times IFS^{-1.3102}\times SFF^{1.7745}. \end{array}$$

## 4. Results

In this section, the experimental numerical results are presented. First, the used benchmark IQA databases are introduced in Section 4.1. Next, Section 4.2 defines the applied evaluation metrics. A parameter study with respect to the applied optimization methods is presented in Section 4.3. Finally, the results of a comparison to the state of the art is given in Section 4.4.

### 4.1. Databases

For evaluation, four IQA benchmark databases are used, i.e., LIVE (Laboratory for Image and Video Engineering) [4], TID2013 (Tampere Image Database) [76], TID2008 [77], and CSIQ (Categorical Image Quality) [33], which contain a small set of reference images (whose perceptual qualities are considered perfect) and a large set of quality annotated distorted images generated from the reference images using different distortion types at different distortion levels. The main characteristics of the applied databases are given in Table 2.

### 4.2. Evaluation Metrics

In this study, four different performance indices, i.e., root mean square error (RMSE), Pearson’s linear correlation coefficient (PLCC), Spearman’s rank-order correlation coefficient (SROCC), and Kendall’s rank-order correlation coefficient (KROCC), are applied to characterize the performance of the proposed fusion-based metric and other considered state-of-the-art methods in an ablation study and a comparison to the state of the art. The RMSE and PLCC are calculated after a non-linear mapping of the vector of predicted scores. This mapping has already been given by Equation (8). The RMSE is given as

(21) $$RMSE\left(Q_{p},S\right)=\sqrt{\frac{{\left(Q_{p}-S\right)}^{T}\left(Q_{p}-S\right)}{m}}$$

where $Q_{p}$ is the vector of predicted scores after the non-linear mapping, S is the vector of ground-truth scores, and *m* denotes the number of samples (in this case images). PLCC is given as

(22) $$PLCC\left(Q_{p},S\right)=\frac{{\bar{Q}}_{p}^{T}\bar{S}}{\sqrt{{\bar{Q}}_{p}^{T}{\bar{Q}}_{p}{\bar{S}}^{T}\bar{S}}}$$

where ${\bar{Q}}_{p}$ and $\bar{S}$ are mean-removed vectors. SROCC is defined as

(23) $$SROCC\left(Q,S\right)=1-\frac{6\times \sum_{i=1}^{m}d_{i}^{2}}{m(m^{2}-1)},$$

where $d_{i}$ stands for the difference between Q and S at the *i*th entry. KROCC is defined as

(24) $$KROCC\left(Q,S\right)=\frac{m_{c}-m_{d}}{\frac{1}{2}m\left(m-1\right)},$$

where $m_{c}$ and $m_{d}$ are the number of concordant and discordant pairs in the database, respectively.

In Table 3, the details of the computer configuration applied in our experiments are given.

### 4.3. Ablation Study

In this subsection, an ablation study is carried out with respect to the applied optimization method. As already mentioned, a GA [70], a PS [71], and the simplex method of Lagarias et al. [72] (implemented in MATLAB’s fminsearch) were considered. Further, the obtained metrics have already been given by Equations (9)–(20). The results in terms of the RMSE and SROCC are summarized in Table 4 and Table 5. From these numerical results, it can be clearly seen that the weights obtained by the GA are significantly better than those of the other two methods. Namely, they provide a consistently good performance in terms of the RMSE and SROCC on all the databases, while the other weights fail to provide a good performance in several cases and sometimes give an unacceptably high RMSE or a low SROCC (the underlined values in Table 4).

### 4.4. Comparison to the State of the Art

In this subsection, the proposed FR-IQA metrics are compared to a set of state-of-the-art methods (2stepQA [78], CSV [79], DISTS [80], ESSIM [24], FSIM [39], FSIMc [39], GSM [63], IFC [9], IFS [81], IW-SSIM [26], MAD [33], MS-SSIM [8], NQM [64], PSNR [21], ReSIFT [82], RFSIM [37], RVSIM [83], SFF [65], SR-SIM [66], SSIM [13], SUMMER [84], VIF [62], and VSI [68]), whose MATLAB source codes were made available by researchers. Further, we reimplemented the SSIM-CNN method proposed by Amirshahi et al. [16] and made it available for the research community (https://github.com/Skythianos/SSIM-CNN, accessed on 1 January 2023). In addition to this, the results of the recently published GP-SSIM [85] and the results of deep learning-based DeepSIM [86], DeepIQA [44], PieAPP [46], and LPIPS [45] are also included in our comparison based on Bakurov et al.’s [85] study. Our proposed fusion-based FR-IQA metrics have also been exactly given by Equations (9)–(20) and are used in this comparison.

**Figure 3:**
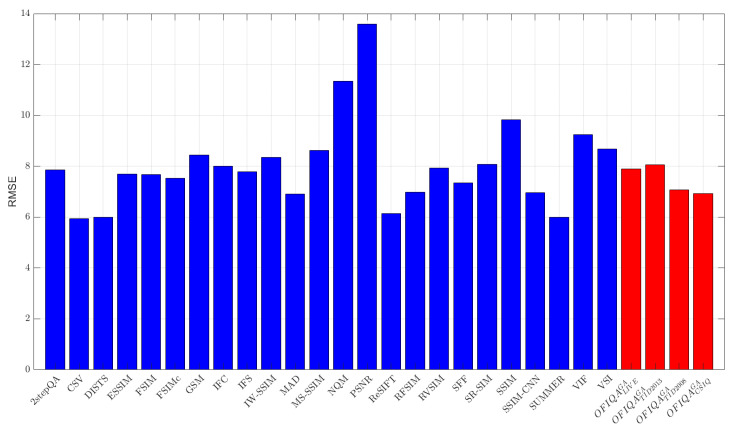
RMSE measured on LIVE [4].

**Figure 4:**
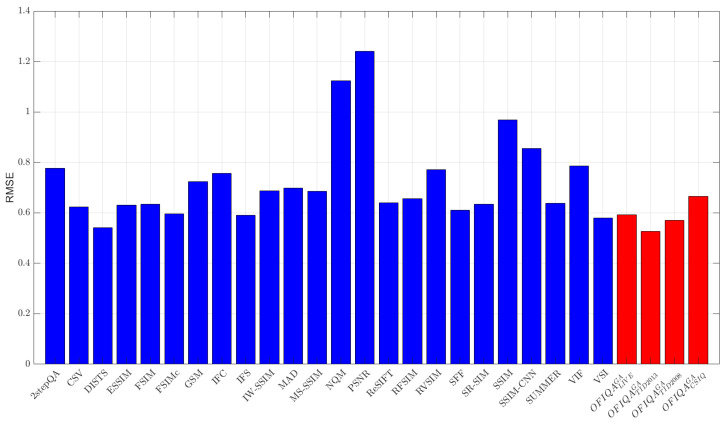
RMSE measured on TID2013 [76].

**Figure 5:**
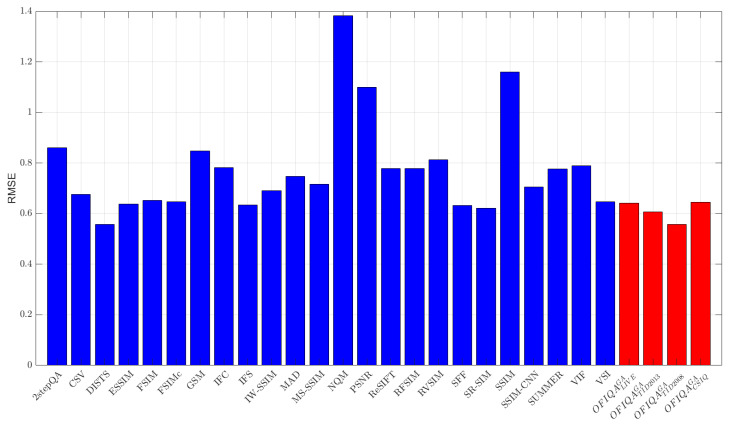
RMSE measured on TID2008 [77].

**Figure 6:**
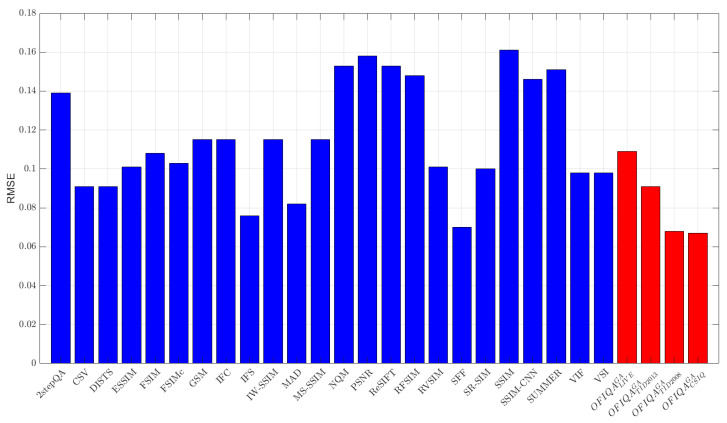
RMSE measured on CSIQ [33].

The numerical results measured on different databases are summarized in Table 6, Table 7 and Table 8. From these results, it can be seen that $OFIQA_{TID2013}^{GA}$ is able to provide the lowest error on TID2013 [76] and the second lowest on TID2008 [77], respectively. On the other hand, $OFIQA_{TID2008}^{GA}$ gives the lowest, second lowest, and third lowest error on TID2008 [77], CSIQ [33], and TID2013 [76], respectively. The RMSE values for each database are summarized in Figure 3, Figure 4, Figure 5 and Figure 6. If we take a look at the correlation strengths, we can observe the following. On LIVE [4], the deep learning-based DeepSIM provides the highest correlation performance. However, the proposed $OFIQA_{CSIQ}^{GA}$’s results closely follow those of DeepSIM. Namely, the differences between the two methods are 0.01 and 0.02 in terms of PLCC and SROCC, respectively. Further, $OFIQA_{TID2008}^{GA}$ provides the third highest results in terms of PLCC and SROCC on CLIVE [4]. On TID2013 [76], $OFIQA_{TID2013}^{GA}$ gives the highest results in terms of PLCC and KROCC, while $OFIQA_{TID2008}^{GA}$ provides the third highest PLCC. On TID2008 [77], $OFIQA_{TID2008}^{GA}$ has the highest SROCC and KROCC values and it is outperformed by PLCC in the deep learning-based DeepIQA. Table 9 illustrates a summary of the direct and weighted averages of the correlation performance indices on the considered databases. It can be seen that $OFIQA_{TID2008}^{GA}$ has the highest performance in terms of PLCC and KROCC, if we consider the direct averages of the correlation strengths. $OFIQA_{TID2008}^{GA}$ preserves it first in terms of PLCC and gives the second highest performance of KROCC, if we consider the weighted averages. $OFIQA_{TID2013}^{GA}$ is the second best in terms of PLCC/SROCC and the third best in terms of KROCC. In the weighted averages, the VSI [68] is the best in terms of SROCC and the third best in terms of PLCC/KROCC. However, it gives a higher RMSE on the considered IQA databases than $OFIQA_{TID2013}^{GA}$ which is the second best weighted SROCC.

## 5. Conclusions

This paper proposed a novel decision-fusion framework based on optimization for FR-IQA. First, the fusion-based metric was defined as a weighted product of *n* different traditional FR-IQA measures following Okarma’s [7] ideas. Next, an optimization problem was specified using SROCC, KROCC, and the RMSE between the predicted and the ground-truth quality scores in the objective function to maximize the correlation strength and minimize the error. Then, several optimization techniques were applied to determine the weights in the weighted product of quality measures. To obtain a fusion-based metric with its parameters, 20% of the reference images and their distorted counterparts were used. Our analysis revealed that a GA is a suitable choice to solve the defined optimization problem. The experimental results and a comparison to the state of the art on four large, widely accepted benchmark databases, LIVE [4], TID2013 [76], TID2008 [77], and CSIQ [33], uncovered that the FR-IQA metrics coming from our optimization-based framework are able to outperform other traditional and deep learning-based state-of-the-art algorithms. Future research may involve the usage of other objective functions or multi-objective optimization. 

## Figures and Tables

**Table 1:** Acronyms of the used FR-IQA metrics applied in our fusion-based method.

Method’s Acronym	Full Name
FSIM [39]	feature similarity index
FSIMc [39]	feature similarity index extension
GSM [63]	gradient similarity measure
IFC [9]	information fidelity criterion
IW-SSIM [26]	information content weighted SSIM
MAD [33]	most apparent distortion
MS-SSIM [8]	multi-scale SSIM
NQM [64]	noise quality measure
PSNR [21]	peak signal-to-noise ratio
RFSIM [37]	Riesz-transform-based feature similarity metric
SFF [65]	sparse feature fidelity
SSIM [13]	structural similarity index measure
SR-SIM [66]	spectral residual-based similarity
UQI [67]	universal image quality index
VIF [62]	visual information fidelity
VSI [68]	visual saliency-induced index
VSNR [69]	visual signal-to-noise ratio

**Table 2:** Applied benchmark IQA databases.

	LIVE [4]	TID2013 [76]	TID2008 [77]	CSIQ [33]
Image resolution	∼768×512	512×384	512×384	500×500
No. of reference images	29	25	25	30
No. of distorted images	779	3000	1700	866
No. of distortions	5	24	17	6
No. of levels	5	5	4	4–5
No. of observers	161	917	838	35

**Table 3:** Computer configuration applied in our experiments.

Computer model	STRIX Z270H Gaming
Operating system	Windows 10
Memory	15 GB
CPU	Intel(R) Core(TM) i7-7700K CPU 4.20 GHz (8 cores)
GPU	Nvidia GeForce GTX 1080

**Table 4:** RMSE performance comparison with respect to the applied optimization method applied in the proposed fusion-based FR-IQA metrics. Results are given for LIVE [4], TID2013 [76], TID2008 [77], and CSIQ [33]. The best results for each database are typed in red, the second best results are in green, the third best results are in blue, and the worst results are underlined.

FR-IQA Metric	LIVE [4]	TID2013 [76]	TID2008 [77]	CSIQ [33]
$OFIQA_{LIVE}^{GA}$	7.895	0.593	0.641	0.109
$OFIQA_{TID2013}^{GA}$	8.062	0.526	0.606	0.091
$OFIQA_{TID2008}^{GA}$	7.078	0.570	0.557	0.068
$OFIQA_{CSIQ}^{GA}$	6.918	0.665	0.645	0.067
$OFIQA_{LIVE}^{PS}$	6.860	0.675	1.116	0.165
$OFIQA_{TID2013}^{PS}$	7.503	1.150	0.537	0.069
$OFIQA_{TID2008}^{PS}$	7.662	0.766	0.544	0.081
$OFIQA_{CSIQ}^{PS}$	7.882	0.658	0.672	0.124
$OFIQA_{LIVE}^{fmin}$	6.612	0.642	0.648	0.060
$OFIQA_{TID2013}^{fmin}$	16.195	0.856	0.834	0.833
$OFIQA_{TID2008}^{fmin}$	14.456	0.833	0.631	0.630
$OFIQA_{CSIQ}^{fmin}$	7.167	0.812	0.680	0.104

**Table 5:** SROCC performance comparison with respect to the applied optimization method applied in the proposed fusion-based FR-IQA metrics. Results are given for LIVE [4], TID2013 [76], TID2008 [77], and CSIQ [33]. The best results for each database are typed in red, the second best results are in green, the third best results are in blue, and the worst results are underlined.

FR-IQA Metric	LIVE [4]	TID2013 [76]	TID2008 [77]	CSIQ [33]
$OFIQA_{LIVE}^{GA}$	0.961	0.863	0.888	0.938
$OFIQA_{TID2013}^{GA}$	0.957	0.890	0.904	0.923
$OFIQA_{TID2008}^{GA}$	0.967	0.825	0.911	0.964
$OFIQA_{CSIQ}^{GA}$	0.972	0.808	0.882	0.965
$OFIQA_{LIVE}^{PS}$	0.968	0.790	0.585	0.807
$OFIQA_{TID2013}^{PS}$	0.965	0.826	0.914	0.962
$OFIQA_{TID2008}^{PS}$	0.963	0.822	0.915	0.944
$OFIQA_{CSIQ}^{PS}$	0.965	0.794	0.861	0.964
$OFIQA_{LIVE}^{fmin}$	0.972	0.864	0.867	0.969
$OFIQA_{TID2013}^{fmin}$	0.781	0.845	0.769	0.496
$OFIQA_{TID2008}^{fmin}$	0.834	0.717	0.888	0.586
$OFIQA_{CSIQ}^{fmin}$	0.974	0.823	0.846	0.955

**Table 6:** RMSE performance comparison of the proposed fusion-based FR-IQA metrics with the state of the art on LIVE [4], TID2013 [76], TID2008 [77], and CSIQ [33]. The best results are typed in red, the second best results are in green, and the third best results are in blue.

FR-IQA Metric	LIVE [4]	TID2013 [76]	TID2008 [77]	CSIQ [33]
2stepQA [78]	7.856	0.776	0.861	0.139
CSV [79]	5.945	0.624	0.677	0.091
DISTS [80]	6.005	0.541	0.558	0.091
ESSIM [24]	7.689	0.630	0.638	0.101
FSIM [39]	7.678	0.635	0.653	0.108
FSIMc [39]	7.530	0.596	0.647	0.103
GSM [63]	8.433	0.723	0.847	0.115
IFC [9]	8.001	0.756	0.783	0.115
IFS [81]	7.776	0.591	0.635	0.076
IW-SSIM [26]	8.347	0.688	0.690	0.115
MAD [33]	6.907	0.698	0.747	0.082
MS-SSIM [8]	8.619	0.686	0.717	0.115
NQM [64]	11.347	1.123	1.382	0.153
PSNR [21]	13.596	1.240	1.099	0.158
ReSIFT [82]	6.145	0.640	0.779	0.153
RFSIM [37]	6.989	0.657	0.778	0.148
RVSIM [83]	7.927	0.772	0.813	0.101
SFF [65]	7.346	0.610	0.633	0.070
SR-SIM [66]	8.081	0.635	0.621	0.100
SSIM [13]	9.831	0.968	1.160	0.161
SSIM-CNN [16]	6.967	0.856	0.705	0.146
SUMMER [84]	6.002	0.638	0.777	0.151
VIF [62]	9.240	0.786	0.790	0.098
VSI [68]	8.681	0.580	0.647	0.098
GP-SSIM [85]	-	-	-	-
DeepSIM [86]	-	-	-	-
DeepIQA [44]	-	-	-	-
PieAPP [46]	-	-	-	-
LPIPS [45]	-	-	-	-
$OFIQA_{LIVE}^{GA}$	7.895	0.593	0.641	0.109
$OFIQA_{TID2013}^{GA}$	8.062	0.526	0.606	0.091
$OFIQA_{TID2008}^{GA}$	7.078	0.570	0.557	0.068
$OFIQA_{CSIQ}^{GA}$	6.918	0.665	0.645	0.067

**Table 7:** PLCC, SROCC, and KROCC performance comparison of the proposed fusion-based FR-IQA metrics with the state of the art on LIVE [4] and TID2013 [76]. The best results are typed in red, the second best results are in green, and the third best results are in blue.

	LIVE [4]			TID2013 [76]		
FR-IQA Metric	PLCC	SROCC	KROCC	PLCC	SROCC	KROCC
2stepQA [78]	0.937	0.932	0.828	0.736	0.733	0.550
CSV [79]	0.967	0.959	0.834	0.852	0.848	0.657
DISTS [80]	0.954	0.954	0.811	0.759	0.711	0.524
ESSIM [24]	0.963	0.962	0.840	0.740	0.797	0.627
FSIM [39]	0.960	0.963	0.833	0.859	0.802	0.629
FSIMc [39]	0.961	0.965	0.836	0.877	0.851	0.667
GSM [63]	0.944	0.955	0.831	0.789	0.787	0.593
IFC [9]	0.927	0.926	0.758	0.554	0.539	0.394
IFS [81]	0.959	0.960	0.825	0.879	0.870	0.679
IW-SSIM [26]	0.952	0.956	0.817	0.832	0.778	0.598
MAD [33]	0.967	0.967	0.842	0.827	0.778	0.600
MS-SSIM [8]	0.941	0.951	0.804	0.794	0.785	0.604
NQM [64]	0.912	0.909	0.741	0.690	0.643	0.474
PSNR [21]	0.872	0.876	0.687	0.616	0.646	0.467
ReSIFT [82]	0.961	0.962	0.838	0.630	0.623	0.471
RFSIM [37]	0.935	0.940	0.782	0.833	0.774	0.595
RVSIM [83]	0.641	0.630	0.495	0.763	0.683	0.520
SFF [65]	0.963	0.965	0.836	0.871	0.851	0.658
SR-SIM [66]	0.955	0.962	0.829	0.859	0.800	0.631
SSIM [13]	0.941	0.951	0.804	0.618	0.616	0.437
SSIM-CNN [16]	0.965	0.963	0.838	0.759	0.752	0.566
SUMMER [84]	0.967	0.959	0.833	0.623	0.622	0.472
VIF [62]	0.941	0.964	0.828	0.774	0.677	0.515
VSI [68]	0.948	0.952	0.805	0.900	0.894	0.677
GP-SSIM [85]	0.908	0.918	-	0.846	0.808	-
DeepSIM [86]	0.968	0.974	-	0.872	0.846	-
DeepIQA [44]	0.940	0.947	-	0.834	0.831	-
PieAPP [46]	0.908	0.919	-	0.859	0.876	-
LPIPS [45]	0.932	0.934	-	0.749	0.670	-
$OFIQA_{LIVE}^{GA}$	0.957	0.961	0.828	0.878	0.863	0.672
$OFIQA_{TID2013}^{GA}$	0.956	0.957	0.814	0.906	0.890	0.713
$OFIQA_{TID2008}^{GA}$	0.966	0.967	0.839	0.888	0.825	0.651
$OFIQA_{CSIQ}^{GA}$	0.967	0.972	0.854	0.844	0.808	0.634

**Table 8:** PLCC, SROCC, and KROCC performance comparison of the proposed fusion-based FR-IQA metrics with the state of the art on TID2008 [77] and CSIQ [33]. The best results are typed in red, the second best results are in green, and the third best results are in blue.

	TID2008 [77]			CSIQ [33]		
FR-IQA Metric	PLCC	SROCC	KROCC	PLCC	SROCC	KROCC
2stepQA [78]	0.757	0.769	0.574	0.841	0.849	0.655
CSV [79]	0.852	0.848	0.657	0.933	0.933	0.766
DISTS [80]	0.705	0.668	0.488	0.930	0.930	0.764
ESSIM [24]	0.658	0.876	0.696	0.814	0.933	0.768
FSIM [39]	0.874	0.881	0.695	0.912	0.924	0.757
FSIMc [39]	0.876	0.884	0.699	0.919	0.931	0.769
GSM [63]	0.782	0.781	0.578	0.896	0.911	0.737
IFC [9]	0.575	0.568	0.424	0.837	0.767	0.590
IFS [81]	0.879	0.869	0.678	0.958	0.958	0.817
IW-SSIM [26]	0.842	0.856	0.664	0.804	0.921	0.753
MAD [33]	0.831	0.829	0.639	0.950	0.947	0.797
MS-SSIM [8]	0.838	0.846	0.648	0.899	0.913	0.739
NQM [64]	0.608	0.624	0.461	0.743	0.740	0.564
PSNR [21]	0.447	0.489	0.346	0.853	0.809	0.599
ReSIFT [82]	0.627	0.632	0.484	0.884	0.868	0.695
RFSIM [37]	0.865	0.868	0.678	0.912	0.930	0.765
RVSIM [83]	0.789	0.743	0.566	0.923	0.903	0.728
SFF [65]	0.871	0.851	0.658	0.964	0.960	0.826
SR-SIM [66]	0.859	0.799	0.631	0.925	0.932	0.773
SSIM [13]	0.669	0.675	0.485	0.812	0.812	0.606
SSIM-CNN [16]	0.770	0.737	0.551	0.952	0.946	0.794
SUMMER [84]	0.817	0.823	0.623	0.826	0.830	0.658
VIF [62]	0.808	0.749	0.586	0.928	0.920	0.754
VSI [68]	0.898	0.896	0.709	0.928	0.942	0.785
GP-SSIM [85]	0.859	0.892	-	0.928	0.953	-
DeepSIM [86]	0.876	0.887	-	0.919	0.919	-
DeepIQA [44]	0.917	0.908	-	0.901	0.909	-
PieAPP [46]	0.610	0.788	-	0.877	0.892	-
LPIPS [45]	0.772	0.731	-	0.896	0.876	-
$OFIQA_{LIVE}^{GA}$	0.879	0.888	0.700	0.910	0.938	0.786
$OFIQA_{TID2013}^{GA}$	0.892	0.904	0.722	0.938	0.923	0.754
$OFIQA_{TID2008}^{GA}$	0.910	0.911	0.738	0.966	0.964	0.833
$OFIQA_{CSIQ}^{GA}$	0.877	0.882	0.693	0.967	0.965	0.835

**Table 9:** PLCC, SROCC, and KROCC performance comparison of the proposed fusion-based FR-IQA metrics with the state of the art. The best results are typed in red, the second best results are in green, and the third best results are in blue.

	Direct Average			Weighted Average		
FR-IQA Metric	PLCC	SROCC	KROCC	PLCC	SROCC	KROCC
2stepQA [78]	0.818	0.821	0.652	0.781	0.783	0.605
CSV [79]	0.901	0.897	0.729	0.877	0.873	0.694
DISTS [80]	0.837	0.816	0.647	0.792	0.759	0.582
ESSIM [24]	0.794	0.892	0.733	0.756	0.857	0.691
FSIM [39]	0.901	0.893	0.729	0.883	0.860	0.689
FSIMc [39]	0.908	0.908	0.743	0.893	0.885	0.710
GSM [63]	0.853	0.859	0.685	0.821	0.823	0.638
IFC [9]	0.723	0.700	0.542	0.644	0.625	0.473
IFS [81]	0.919	0.914	0.750	0.900	0.893	0.715
IW-SSIM [26]	0.857	0.878	0.708	0.846	0.840	0.664
MAD [33]	0.894	0.880	0.720	0.862	0.838	0.667
MS-SSIM [8]	0.868	0.874	0.699	0.838	0.839	0.659
NQM [64]	0.738	0.729	0.560	0.703	0.684	0.516
PSNR [21]	0.697	0.705	0.525	0.634	0.654	0.480
ReSIFT [82]	0.776	0.771	0.622	0.705	0.700	0.550
RFSIM [37]	0.886	0.878	0.705	0.865	0.841	0.663
RVSIM [83]	0.779	0.740	0.577	0.777	0.723	0.558
SFF [65]	0.917	0.908	0.745	0.895	0.880	0.703
SR-SIM [66]	0.900	0.873	0.716	0.880	0.838	0.675
SSIM [13]	0.760	0.764	0.583	0.698	0.700	0.518
SSIM-CNN [16]	0.861	0.849	0.687	0.814	0.800	0.626
SUMMER [84]	0.808	0.809	0.647	0.745	0.746	0.582
VIF [62]	0.863	0.828	0.671	0.825	0.765	0.605
VSI [68]	0.919	0.921	0.744	0.909	0.908	0.716
GP-SSIM [85]	0.885	0.893	-	0.868	0.864	-
DeepSIM [86]	0.909	0.907	-	0.891	0.883	-
DeepIQA [44]	0.898	0.899	-	0.878	0.877	-
PieAPP [46]	0.814	0.869	-	0.801	0.860	-
LPIPS [45]	0.837	0.803	-	0.798	0.747	-
$OFIQA_{LIVE}^{GA}$	0.906	0.913	0.747	0.892	0.892	0.714
$OFIQA_{TID2013}^{GA}$	0.923	0.919	0.751	0.913	0.906	0.733
$OFIQA_{TID2008}^{GA}$	0.933	0.917	0.765	0.914	0.884	0.722
$OFIQA_{CSIQ}^{GA}$	0.914	0.907	0.754	0.885	0.869	0.704

## Data Availability

In this paper, the following publicly available benchmark databases were used: 1. LIVE: https://live.ece.utexas.edu/research/quality/subjective.htm (accessed on 12 April 2023), 2. TID2013: http://www.ponomarenko.info/tid2013.htm (accessed on 12 April 2023), 3. TID2008: http://www.ponomarenko.info/tid2008.htm (accessed on 12 April 2023), and 4. CSIQ: https://isp.uv.es/data_quality.html (accessed on 12 April 2023).

## Abbreviations

The following abbreviations are used in this manuscript:

CNN	convolutional neural network
CPU	central processing unit
CQM	combined quality metric
CSIQ	categorical image quality
FR	full reference
FR-IQA	full-reference image quality assessment
GA	genetic algorithm
GPU	graphics processing unit
HIK	histogram intersection kernel
HVS	human visual system
IQA	image quality assessment
KROCC	Kendall’s rank-order correlation coefficient
LIVE	laboratory for image and video engineering
NR	no reference
PLCC	Pearson’s linear correlation coefficient
PS	pattern search
RMSE	root mean square error
RR	reduced reference
SROCC	Spearman’s rank-order correlation coefficient
SSIM	structural similarity index
TID	Tampere image database
